# Social-support needs among adolescents living with HIV in transition from pediatric to adult care in Cambodia: findings from a cross-sectional study

**DOI:** 10.1186/s12981-018-0195-x

**Published:** 2018-03-28

**Authors:** Graham Toth, Gitau Mburu, Sovannary Tuot, Vohith Khol, Chanrith Ngin, Pheak Chhoun, Siyan Yi

**Affiliations:** 1 Fielding School of Public Health, University of California Los Angeles, Los Angeles, USA; 2 Division of Health Research, Lancaster University, Lancaster, UK; 3 KHANA Center for Population Health Research, No. 33, Street 71, Phnom Penh, Cambodia; 4 National Center for HIV/AIDS, Dermatology and STD, Phnom Penh, Cambodia; 5 Center for Global Health Research, Touro University California, Vallejo, USA

**Keywords:** HIV, Adolescents, Transition, Social support, Social protection, Cambodia

## Abstract

**Background:**

Understanding the circumstances of adolescents living with HIV is critical in designing adolescent-friendly services that will facilitate successful transition from pediatric to adult care. This study describes access, utilization and ongoing social support needs among adolescents living with HIV aged 15–17 in transition from pediatric to adult HIV care in Cambodia.

**Methods:**

A cross-sectional study was conducted among 328 adolescents, randomly selected from 11 antiretroviral therapy (ART) clinics across the country. Descriptive analyses were conducted to summarize their characteristics, access to social support and ongoing support needs among male and female adolescents.

**Results:**

Mean age of the study participants was 15.8 (SD = 0.8) years. Just over half (55.2%) were male. Most had at least one deceased parent (mother 50.9%; father 60.5%), and majority were living with biological parents (40.8%) or relatives (49.3%). A third came from families with an ID poor card, and 21.0% were working for pay. Almost half (46.6%) reported that their family had received social support for their health care, including food support (76.5%), school allowance (62.1%), transport allowance to ART clinics (53.6%), psychosocial counseling (35.3%), vocational training (22.9%) or home visits (11.1%). Several ongoing social support needs were identified, including ongoing inability to cover health expenses unless they are supported by health insurance or health equity fund (55.0%). In addition, adolescents reported having been asked to come back earlier than their scheduled appointment (13.7%), having had to purchase their own drugs (2.7%), experiencing HIV stigma (32.0%), having been denied housing or food due to HIV (8.2%) or failing to attend school within the past month partly because of HIV (16.8%). Two-thirds did not have access to peer support groups.

**Conclusions:**

Social protection mechanisms are reaching some adolescents in need, while other remain without social support due to discontinuities in health and social care. Multi-sectoral interventions, supporting school attendance, adolescent-friendly clinic scheduling, reductions in child employment, mitigation of HIV-related stigma and strengthening of peer-to-peer support are required to improve coverage of social protection interventions for adolescents in transition.

## Background

Adolescents, defined as people aged 10–19 years, undergo rapid psychological, sexual and physical maturity [1]. Adolescents experience marked changes in sexuality [2], mental capacity [3], self-efficacy and independence [4]. At the same time, adolescents are one of the most affected populations by HIV. Globally, over two million adolescents are living with HIV [5]. Given the dynamic changes during adolescence, studies exploring outcomes of HIV care tend to distinguish adolescents living with HIV from both pediatric and adult populations.

Evidence from these studies shows that adolescents’ access and outcomes of care are generally sub-optimal. Compared to adults, adolescents have poorer rates of HIV testing, disclosure [6], treatment adherence [7], long-term immunologic recovery [8] and viral suppression [9, 10]. In addition, adolescents often face denial of care by their legal guardians and parents [11], are inexperienced in negotiation for safer sex during sexual debut [12, 13] and may be unable to obtain services due to restrictive parental consent laws [14]. Exploration of causes and potential solutions to these poor outcomes have generally focused on biomedical and health system solution [15].

However, the issues of social support for adolescents living with HIV are increasingly occupying a center stage. Due to the increasing survival of children living with HIV into adolescence [16], some of whom were orphans or had lost their primary caregivers [17, 18], there is rising recognition that they require social support to adjust socially and psychologically to living with HIV [19, 20].

The influence of social support on adolescent’s self-efficacy [4], uptake of new services, acceptance of their diagnosis and treatment adherence [21, 22] have been documented in recent studies. Additionally, peer support groups have been shown to have a positive impact on disclosure, mental health [23] and uptake of HIV services [24] among adolescents. Furthermore, structural social protection interventions—such as cash transfers, food security, school fees and feeding programs—have been shown to have a positive impact on adolescents’ adherence to antiretroviral therapy (ART) and general wellbeing [25].

These findings suggest that social support and protection are essential in achieving positive health outcomes among adolescents living with HIV. However, adolescents are not a homogenous group, but instead, they have evolving social needs in tandem with their physical and psychological maturity [26]. Studies suggest that older adolescents are particularly at risk of poorer HIV outcomes during their transition from pediatric to adult HIV care [27]. During this period, adolescents living with HIV are at high risk of poor adherence to ART [28], disengagement from HIV care [29], immunological deterioration [30] and death [5, 31].

However, there is a paucity of research exploring social support during adolescents’ transition from pediatric to adult HIV care. Most of the existing studies tend to focus on clinical and biomedical (e.g., virological, retention and mortality) outcomes during and after transition to adult care [28, 29, 32, 33], with little focus on the social support interventions or needs specific to this group. As a result, social support for adolescents living with HIV remain undocumented and unaddressed [30]. This paper describes utilization and ongoing social support needs among adolescents living with HIV aged 15–17 in transition from pediatric to adult care in Cambodia and discusses the implications of the findings on a successful transition from pediatric to adult HIV care.

## Methods

### Study design and settings

This cross-sectional study was conducted in August 2016 among 328 adolescents living with HIV, randomly selected from 11 ART clinics throughout Cambodia. The details of the study have been reported elsewhere [34]. Briefly, we applied a two-stage sampling design to identify 598 eligible participants from the 18 major ART clinics countrywide. Twenty-one clinics with less than 10 adolescents living with HIV each were excluded to minimize cost. The sample size was set at 320, calculated to estimate a high level of preparedness of 60% with a confidence interval (CI) of 95% and design effect of 1.4 using a probability proportional to size method [35]. Eligible subjects were selected using a random number table.

### Participant recruitment

Selected adolescents were screened via telephone and informed of research objectives as well as place, date and time of the interview. Adolescents would be asked to participate if they were between 15 and 17 years of age, receiving treatment and care services, able to communicate in Khmer, allowed by a parent or guardian to participate, their parent or caregiver was willing to provide a written consent, able to present themselves on the day of the interview and physically and mentally stable to provide verbal assent to participate. Upon confirming eligibility, a written consent from their parent or guardian was obtained. Recruitment continued until the required sample size for each clinic was achieved.

### Questionnaire development

Questionnaire development was guided by existing literature [36, 37]. Information on socio-demographic characteristics, perceived health status, school attendance, disclosure, stigma experience, psychosocial support and medication supply and ART adherence were collected through self-reported interviews. Clinical and immunological data, including HIV status, ART history and laboratory values for CD4 and viral loads were obtained from medical records.

### Data collection and training

Data were collected by two teams of interviewers and moderators who were trained on study protocol, questionnaire administration, confidentiality and issues related to research with human participants [38]. Pre-testing was conducted among 20 adolescents living with HIV and 10 parents and guardians, and questionnaire adjustments were made.

### Data analyses

Data were classified and scaled continuously or categorically as appropriate and recorded into a computerized database using EpiData version 3 (Odense, Denmark). Double data entry was implemented to minimize errors. Descriptive analyses were performed to determine frequency distribution for categorical variables and mean with standard deviation (SD) for continuous variables. Chi square, Fisher’s exact or t-test tests for significance were used to compare characteristics of male and female adolescents. p-values of less than 0.05 were considered statistically significant.

### Ethical considerations

The study was approved by the National Ethics Committee for Health Research, Ministry of Health in Cambodia (Ref: 297NECHR). Assent from participants and a written informed consent from a parent or guardian was obtained. Data were collected in private locations, and confidentiality protected by removing all personal identifiers from questionnaires. Participants were provided with a token of $2.5–$5 for transportation.

## Results

### Characteristics and of the study sample

Of the 328 adolescents, 51.3% had completed secondary (7–9 years) school, less than half (42.7%) were living with one or both parents, and 55.2% were male. Parents (40.8%) or relatives (33.6%) were the most frequently reported caregivers. More than half of respondents had at least one deceased parent (mother 50.9%; father 60.5%). Maternal and paternal educational levels were both 20% for high school (10–12 years) or university education (Table 1).

**Table 1:** Socio-demographic characteristics of male and female adolescents living with HIV

Socio-demographic characteristics	Total (*n *= 328), *n* (%)	Male (*n *= 169), *n* (%)	Female (*n *= 159), *n* (%)	*P*-value*
Mean age (in years)	15.9 ± 0.8	15.8 ± 0.8	16.0 ± 0.8	0.06
Type of ART regimen				0.91
First line	183 (82.4)	140 (82.8)	131 (82.4)	
Second line	36 (16.2)	29 (17.2)	27 (17.6)	
Duration on ART (in months)	101 ± 40	99.8 (40.7)	94.0 (39.6)	0.20
Initial CD4 count (cells/mm^3^)	632 ± 110	777 ± 1460	632 ± 460	0.35
Latest CD4 count (cells/mm^3^)	672 ± 284	683 ± 314	660 ± 250	0.44
First viral load count (copies)	34,375 ± 13,958	37,868 ± 13,205	30,662 ± 14,748	0.64
Latest viral load count (copies)	9268 ± 6407	4103 ± 1573	14,759 ± 9041	0.13
Visual adherence scale (%)	95.4 ± 9.6	95.3 ± 9.6	95.4 ± 9.7	0.91
Travel time to ART clinic (min)	48.2 ± 43.4	46.1 ± 41.2	55.5 ± 45.5	0.36
Cost to travel to clinic (USD)	3.4 ± 3.9	3.5 ± 3.9	3.3 ± 4.0	0.61
Level of formal education^a^				0.76
Primary school or lower	92 (28.0)	50 (20.7)	42 (26.4)	
Secondary school	168 (51.3)	86 (50.9)	82 (51.6)	
High school or higher	68 (20.7)	33 (19.5)	35 (22.0)	
Ability to cover health expenses				0.11
No	181 (55.2)	86 (50.9)	95 (59.7)	
Yes	147 (44.8)	83 (49.3)	64 (40.3)	
Family has an ID poor card				0.04
No	190 (57.9)	88 (52.1)	102 (64.2)	
Yes	112 (34.2)	63 (37.3)	49 (30.8)	
Don’t know	26 (7.9)	18 (10.7)	8 (5.0)	
Access to the health equity fund				<0.001
No	185 (56.4)	109 (64.5)	76 (47.8)	
Yes	126 (38.4)	45 (35.7)	81 (50.9)	
Don’t know	17 (5.2)	15 (8.9)	2 (1.3)	
Covered by health insurance				0.02
No	289 (88.1)	148 (87.6)	141 (88.7)	
Yes	148 (87.6)	0 (0.0)	6 (3.8)	
Don’t know	33 (10.1)	21 (12.4)	12 (7.5)	
Currently living with				0.33
Parents	140 (42.7)	73 (43.2)	67 (42.1)	
Grandparents	49 (14.9)	29 (12.7)	20 (12.6)	
Relatives	106 (32.3)	54 (32.0)	52 (32.7)	
In an orphanage	27 (8.2)	12 (7.1)	15 (9.4)	
Other	6 (1.8)	1 (0.6)	5 (3.1)	
Type of accommodation				0.94
Flat/apartment	19 (8.9)	10 (8.7)	9 (8.4)	
Hut	12 (5.4)	7 (6.1)	5 (4.7)	
Mid-size country house	173 (73.7)	88 (78.7)	85 (79.4)	
Mansion/large country house	12 (5.4)	6 (5.2)	6 (5.6)	
Other	6 (2.7)	4 (3.5)	2 (1.9)	
Family possessions				
Running water	123 (55.4)	61 (53.0)	62 (57.9)	0.46
Electricity	195 (87.8)	102 (88.7)	93 (86.9)	0.69
Television	167 (75.2)	88 (76.5)	79 (73.8)	0.64
Radio	77 (34.7)	45 (39.1)	32 (29.9)	0.15
Fridge	30 (13.5)	13 (11.3)	17 (15.9)	0.32
Private latrine	182 (82.0)	96 (83.5)	86 (80.4)	0.55
Mother is still alive				0.18
No	167 (50.9)	80 (47.3)	87 (54.7)	
Yes	161 (49.1)	89 (52.7)	72 (45.3)	
Mother’s education level^a^				0.12
No education	20 (12.7)	7 (8.1)	13 (18.1)	
Primary school	53 (33.5)	27 (31.4)	26 (36.1)	
Secondary school	24 (15.2)	14 (16.3)	10 (13.9)	
High school or higher	24 (15.2)	12 (14.0)	12 (16.7)	
Don’t know	37 (23.4)	26 (30.2)	11 (15.3)	
Father is still alive				0.82
No	198 (60.4)	101 (59.8)	97 (61.0)	
Yes	130 (39.6)	68 (40.2)	62 (39.0)	
Father’s education level^a^				0.04
No education	9 (5.6)	4 (6.2)	3 (4.3)	
Primary school	33 (26.2)	12 (18.8)	21 (33.9)	
Secondary school	18 (14.3)	8 (12.5)	10 (16.1)	
High school or higher	28 (24.3)	9 (14.1)	14 (22.6)	
Don’t know	45 (35.7)	31 (48.4)	14 (22.6)	
Main daily caregiver				0.57
Parent	187 (57.0)	102 (60.4)	85 (53.5)	
Grand parent	5 (1.5)	2 (1.2)	3 (1.9)	
Sibling	24 (7.3)	9 (5.3)	15 (9.4)	
Relatives	100 (30.5)	50 (29.6)	50 (31.4)	
Orphanage/NGO staff	12 (3.7)	6 (3.6)	6 (3.8)	
Main caregiver’s education level^a^				< 0.001
Primary school or lower	14 (7.2)	2 (2.1)	12 (12.1)	
Secondary school	40 (20.5)	8 (8.3)	32 (32.3)	
High school	11 (5.6)	6 (6.2)	5 (5.1)	
University or higher	29 (14.9)	16 (16.7)	13 (13.1)	
Don’t know	101 (51.8)	64 (66.7)	37 (37.4)	

*ART* antiretroviral therapy, *HIV* human immunodeficiency virus, *NGO* non-governmental organization, *USD* United States Dollar

* Chi square or Fisher’s exact test was used as appropriate for categorical variables, Student’s t-test was used for continuous variables

^a^Primary = less than or equal to 6 years, secondary = 7–9 years, high school = 10–12 years

### Access and adherence to ART

On average, ART refills were dispensed every two months. Forty-five participants (13.7%) reported having to return earlier than their scheduled appointment due to inadequate supply of medicines (22.2%) or the need for an additional check-up (46.7%). A small number (2.7%) had been asked to purchase their own drugs. Notably, sharing of ART was not reported (Table 2).

**Table 2:** Access and adherence to ART among male and female adolescents living with HIV

Access and adherence to ART	Total (*n *= 328)	Male (*n *= 169)	Female (*n *= 159)	*p*-value*
	*n* (%)	*n* (%)	*n* (%)	
Mean number of days the drug supply is usually given	58.4 ± 53.9	61.4 ± 73.7	55.1 ± 14.9	0.3
Ever shared your drugs to other persons				–
No	328 (100)	169 (100)	159 (100)	
Yes	0 (0.0)	0 (0.0)	0 (0.0)	
Having been asked to come back earlier than appointment				0.56
No	283 (86.3)	144 (85.2)	139 (87.4)	
Yes	45 (13.7)	25 (14.8)	20 (12.6)	
Reasons for coming back earlier than appointment				0.35
Lack of medicines	10 (22.2)	5 (20.0)	5 (25.0)	
Additional check up	21 (46.7)	10 (40.0)	11 (55.0)	
Other	14 (31.1)	10 (40.0)	4 (20.0)	
Having been asked to buy any other medicines				0.81
No	319 (97.3)	164 (97.0)	155 (97.5)	
Yes	9 (2.7)	5 (3.0)	4 (2.5)	

### Stigma and discrimination

Thirty-two percent reported negative attitudes or being mistreated (10.7%) because of their HIV status; 8.2% had been denied housing or food (Table 3).

**Table 3:** Experiences of stigma and discrimination among male and female adolescents living with HIV

Experiences of stigma and discrimination	Total (*n *= 328)	Male (*n *= 169)	Female (*n *= 159)	*p*-value*
	*n* (%)	*n* (%)	*n* (%)	
Someone has mistreated you because of your HIV-infected status				0.28
No	293 (89.3)	154 (91.1)	139 (87.4)	
Yes	35 (10.7)	15 (8.9)	20 (12.6)	
Having experienced negative attitudes because of your HIV-infected status				0.09
No	223 (68.0)	122 (72.2)	101 (63.5)	
Yes	105 (32.0)	47 (27.8)	58 (36.5)	
Having been refused to share housing/other materials because of your HIV-infected status				0.66
No	301 (91.8)	154 (91.1)	147 (92.5)	
Yes	27 (8.2)	15 (8.9)	12 (7.5)	

### School attendance, employment and social support

Within the past month, 22.9% experienced disruptions with attendance at school, and 16.8% reported that they were no longer enrolled. Being too ill (34.1%), lack of fees (14%) or poor academic performance (18.6%) were the most frequently reported reasons for being absent. Only a fraction (0.6%) of the 69 adolescents currently working for pay sought employment outside of their hometown. Almost half (46.6%) reported that their family had received social support for their health care, including food support (76.5%), school allowance (62.1%), transport allowance to ART clinic (53.6%), emotional counseling (35.3%), vocational training (22.9%) and home visit (11.1%). Thirty percent belonged to a peer support group (Table 4).

**Table 4:** School attendance and employment history among male and female adolescents living with HIV

School attendance and employment history	Total (*n *= 328), *n* (%)	Male (*n *= 169), *n* (%)	Female (*n *= 159), *n* (%)	*p*-value*
School attendance in the past month				0.79
Never	55 (16.8)	29 (17.2)	26 (16.4)	
With disruptions	75 (22.9)	36 (21.3)	39 (24.5)	
Full time	198 (60.4)	104 (61.5)	94 (59.1)	
Age when stopped going to school	14.7 ± 1.5	14.7 ± 1.2	14.6 ± 1.8	0.48
Reasons for not attending school				0.25
No money to go to school	18 (14.0)	6 (9.4)	12 (18.5)	
Too sick to go to school	44 (34.1)	20 (31.2)	24 (36.9)	
Not successful at school	24 (18.6)	15 (23.4)	9 (13.8)	
Other	43 (33.3)	23 (35.9)	20 (30.9)	
Currently working for pay				0.34
No	259 (79.0)	137 (81.1)	122 (76.7)	
Yes	69 (21.0)	32 (18.9)	37 (23.3)	
Mean age when started working	14.8 ± 1.9	15.0 ± 1.2	14.7 ± 2.3	0.54
Ever travelled outside of hometown for work				0.23
No	326 (94.4)	169 (100)	157 (98.7)	
Yes	2 (0.6)	0 (0.0)	2 (1.3)	
Family received social support for your health				0.85
No	175 (53.4)	91 (53.8)	84 (52.8)	
Yes	153 (46.6)	78 (46.2)	75 (47.2)	
Types of support received by family				
Transportation allowance	82 (53.6)	43 (55.1)	39 (52.0)	0.7
Food support	117 (76.5)	57 (73.1)	60 (80.0)	31
School allowance	95 (62.1)	46 (59.0)	49 (65.3)	0.42
Emotional counseling	54 (35.3)	24 (30.8)	30 (40.0)	0.23
Vocational training	35 (22.9)	14 (17.9)	21 (28.0)	0.14
Home visit	17 (11.1)	10 (12.8)	7 (9.3)	0.49
Family received financial support for your health				0.95
No	214 (65.2)	110 (65.1)	104 (65.4)	
Yes	114 (34.8)	59 (34.9)	55 (34.6)	
Belonged to a patient group				0.63
No	231 (70.4)	121 (71.6)	110 (69.2)	
Yes	97 (27.9)	48 (28.4)	49 (30.8)	

*ART* antiretroviral therapy, *HIV* human immunodeficiency virus

* Chi square or Fisher’s exact test was used as appropriate for categorical variables, Student’s t-test was used for continuous variables

### Assessment of preparedness for transition

The majority (72.0%) of respondents were able to recognize when they were getting sick, and 80.5% knew when they needed to call a health care provider. However, less than half (46.0%) reported being the one responsible for scheduling follow-up visits, and 13.4% indicated that they felt comfortable asking health questions during appointments. An approximately equal number of males and females reported some degree of difficulty with ART adherence. The average visual adherence scale score between the two groups was 95.4% (SD = 9.6%) (Table 5).

**Table 5:** Assessment of preparedness for transition from pediatric to adult care among male and female adolescents living with HIV

Assessment of preparedness for transition	Total (*n *= 328), *n* (%)	Male (*n *= 169), *n* (%)	Female (*n *= 159), *n* (%)	*p*-value*
Can you recognize when you are getting sick?				0.28
No	92 (28.0)	43 (25.4)	49 (30.8)	
Yes	236 (72.0)	126 (74.6)	110 (69.2)	
Do you know when you need to call the doctor?				1.00
No	64 (19.5)	33 (19.5)	31 (19.5)	
Yes	264 (80.5)	136 (80.5)	128 (80.5)	
Are you responsible for making your own appointments?				0.40
No	177 (54.0)	95 (56.2)	82 (51.6)	
Yes	151 (46.0)	74 (43.8)	77 (48.4)	
Are you responsible for refilling your own medications?				0.79
No	151 (46.0)	79 (46.7)	72 (45.3)	
Yes	177 (54.0)	90 (53.3)	87 (54.7)	
Do you feel comfortable asking questions at your appointments?				0.04
No	284 (86.6)	140 (82.8)	144 (90.6)	
Yes	44 (13.4)	29 (17.2)	15 (9.4)	
Do you have a copy of your health records and doctor contacts?				< 0.001
No	179 (54.6)	72 (42.6)	107 (67.3)	
Yes	149 (45.4)	97 (57.4)	52 (32.7)	
Do you have a method of keeping track of your healthcare appointments?				< 0.001
No	126 (38.4)	46 (27.2)	80 (50.3)	
Yes	202 (61.6)	123 (72.8)	79 (49.7)	
Do you find it difficult to remember to take your medicine?				0.21
No	250 (76.2)	124 (73.4)	126 (79.2)	
Yes	78 (26.8)	45 (26.6)	33 (20.8)	
When you feel better, do you stop taking your medicine?				0.14
No	326 (99.4)	169 (100)	157 (98.7)	
Yes	2 (0.6)	0 (0.0)	2 (1.3)	
If you feel worse when you take the medicine, do you stop taking it?				0.02
No	292 (89.0)	157 (92.9)	135 (84.9)	
Yes	36 (11.0)	12 (7.1)	24 (15.1)	
Thinking back over the past 4 days, have you missed any of your medicine?				0.81
No	282 (86.0)	147 (87.0)	135 (84.9)	
Not sure	41 (12.5)	20 (11.8)	21 (13.2)	
Yes	5 (1.5)	2 (1.2)	3 (1.9)	
Visual adherence scale (%)	95.4 ± 9.6	95.3 ± 9.6	95.4 ± 9.7	0.91

*HIV* human immunodeficiency virus

Values are number (%) for categorical variables and mean (± SD) for continuous variables

* Chi square or Fisher’s exact test was used for categorical outcome variables and Student’s t-test was used for continuous outcome variables

Significantly, females were less likely to retain copies of their health records and doctor contacts (32.7% vs. 57.4%, *p *< 0.001), less likely to report having a method for organizing HIV-related appointments (47.9% vs. 72.8%, *p *< 0.001) and more likely to report that they would discontinue ART when feeling ill (7.1% vs. 15.1%, *p *= 0.02) (Table 5).

### Experiences of preparation for transition

About one-third (29.6%) had received counseling on the transition to adult services. However, only 2.7% of them had completed a transfer form, and 19.7% had visited an adult ART clinic to prepare for the transition. About one-forth (24.7%) reported that a case manager had been identified to support them during the transition. The majority of them felt supported during the preparation process for transition with 14.5% feeling very supported, and 59.5% felt somewhat supported. Sixty-six percent preferred to receive pediatric HIV-related care. Issues related to health, sexuality or daily life were most frequently discussed with family (47.9%), health providers (20.4%) and friends (13.4%). However, health care providers (79%) were reported as the most trusted source of information for HIV care and treatment (Table 6).

**Table 6:** Experience of transition from pediatric and adult care among male and female adolescents living with HIV

Experience of transition from pediatric and adult care	Total (*n *= 328), *n* (%)	Male (*n *= 169), *n* (%)	Female (*n *= 159), *n* (%)	*p*-value*
Facility you prefer to receive care and treatment				0.46
Pediatric pre ART/ART services	217 (66.2)	115 (68.8)	102 (64.2)	
Adult pre ART/ART service	111 (33.8)	54 (32.0)	57 (35.8)	
Preferred to discuss questions related to health, sexuality, or daily life with				0.01
Health providers	67 (20.4)	45 (26.6)	22 (13.8)	
Counselors/peer educators	19 (5.8)	6 (3.6)	13 (8.2)	
Friends	44 (13.4)	17 (10.1)	27 (17.0)	
Family	157 (47.9)	81 (47.9)	76 (47.8)	
Other	41 (12.5)	20 (11.8)	21 (13.2)	
Person who you trust the most for your treatment				0.02
Health providers	259 (79.0)	132 (78.1)	127 (79.9)	
Counselors/peer educators	14 (4.3)	3 (1.8)	11 (6.9)	
Friends/family	36 (11.0)	25 (14.8)	11 (6.9)	
Other	19 (5.8)	9 (5.8)	10 (6.3)	
Sources of information about health				< 0.001
Pre ART/ART clinic	199 (60.7)	118 (69.8)	81 (50.9)	
NGOs	21 (6.4)	10 (5.9)	11 (6.9)	
Family	80 (24.7)	25 (14.8)	55 (34.6)	
Other	28 (8.5)	16 (9.5)	12 (7.5)	
Current HIV care provider				0.06
Health provider for adults	105 (32.0)	46 (27.2)	59 (37.1)	
Health provider for children	223 (68.8)	123 (72.8)	100 (62.9)	
Received counseling on transition to adult services				0.49
No	190 (57.9)	101 (59.8)	89 (56.0)	
Yes	138 (42.1)	68 (40.2)	70 (44.0)	
Person who provided the counseling				0.34
Health providers	56 (40.9)	31 (45.6)	25 (36.2)	
Counselors/peer educators	64 (46.7)	31 (45.6)	33 (47.8)	
Other	17 (12.4)	6 (8.8)	11 (15.9)	
Ever completed a transfer form				0.58
No	292 (89.0)	152 (89.9)	140 (88.1)	
Yes	36 (11.0)	17 (10.1)	19 (11.9)	
Ever visited an adult clinic to prepare for transition				0.007
No	235 (71.6)	110 (65.1)	125 (78.6)	
Yes	93 (28.4)	59 (34.9)	34 (21.4)	
Person who took you to visit the adult clinic to prepare for transition				0.07
Health providers	8 (8.7)	4 (6.8)	4 (12.1)	
Counselors/peer educators	8 (8.7)	2 (3.4)	6 (18.2)	
Friends/family	58 (63.0)	41 (69.5)	17 (51.5)	
Other	18 (19.6)	12 (20.3)	6 (18.2)	
The visit was helpful for you to cope with the transition				0.83
No	5 (5.6)	3 (5.2)	2 (6.3)	
Yes	85 (94.4)	55 (94.8)	30 (93.8)	
A ‘Case Manager’ has been identified to support you during the transition				0.003
No	192 (58.5)	112 (66.3)	80 (50.3)	
Yes	136 (41.5)	57 (33.7)	79 (49.7)	
Preparedness to manage your treatment going forward				< 0.001
Very prepared	42 (12.8)	27 (16.0)	15 (9.4)	
Somewhat prepared	249 (75.9)	136 (80.5)	113 (71.1)	
Somewhat unprepared	21 (6.4)	4 (2.4)	17 (10.7)	
Very unprepared	16 (4.9)	2 (1.2)	14 (8.8)	
Feeling supported during your transition from pediatric care to adult care				0.006
Very supported	45 (25.4)	17 (20.0)	28 (30.4)	
Somewhat supported	106 (59.9)	49 (57.6)	57 (62.0)	
Somewhat unsupported	13 (7.3)	7 (8.2)	6 (6.5)	
Very unsupported	13 (7.3)	12 (14.1)	1 (1.1)	
Satisfaction you with your transition experience in general				0.34
Very satisfied	53 (16.2)	29 (17.2)	24 (15.1)	
Somewhat satisfied	107 (32.6)	51 (30.2)	56 (35.2)	
Somewhat dissatisfied	34 (10.4)	14 (8.3)	20 (12.6)	
Very dissatisfied	134 (40.7)	75 (44.4)	59 (37.1)	

*ART* antiretroviral therapy, *HIV* human immunodeficiency virus

Values are number (%) for categorical variables and mean (± SD) for continuous variables

* Chi square or Fisher’s exact test was used for categorical outcome variables and Student’s t-test was used for continuous outcome variables

Males were significantly more likely to discuss health problems with medical providers (26.6% vs. 13.8%, *p *= 0.01), and they were also more likely to trust friends or family members the most for their treatment (14.8% vs. 6.9%, *p *= 0.02). Preparation strategies among males were more likely to include a visit to an adult clinic (34.9% vs. 21.4%, *p *= 0.007) despite having a lower likelihood of being assigned a case manager (33.7% vs. 49.7%, *p *= 0.003) or of including counselors or peer-educators in their transition plans (1.8% vs. 6.9%, *p *= 0.02). Overall, male participants reported feeling more prepared for the transition to adult services (96.5% vs. 80.5%, *p *< 0.001), despite being more likely to report feeling inadequately supported (77.6% vs. 92.4%, *p *= 0.006) (Table 6).

## Discussion

Our cross-sectional study describes the dynamic social circumstances unique to Cambodian adolescents living with HIV as they transition to adult clinics. We found that social protection mechanism are reaching some adolescents living with HIV in need. Almost half reported that their family had received at least one element of social support for their health care, ranging from food support, school allowance, transport allowance to ART clinics, psychosocial counseling, vocational training, or home visits. Of these, food support and school allowance had the highest coverage.

At the same time, significant social support needs were identified, including ongoing inability ability to cover health expenses (55.0%), stigma and discrimination and lack of access to peer support groups. Some of these needs intersected with health system factors such as lack of HIV treatment, failure to received counselling on transition, low rate of familiarization with adult clinics and allocation of a transition case manager.

The numbers of participants accessing the social supports indicate that current interventions have the capacity-building potential to reach adolescents living with HIV from poorer communities. This is important given that socioeconomic and social health protection status are significant determinants of a successful move to adult-oriented medical care [30, 39]. Evidence from other setting suggests that both adolescents living with HIV and their health care providers consider poor socioeconomic conditions to be a significant barrier to the transition process [30].

We uncovered remarkable differences in how male and female adolescents living with HIV communicate their health needs and manage their care and treatment. Our data shows that female adolescents have difficulty managing their own care, including discussing their medical concerns with their health care providers, which may explain the more frequent reports of ART non-adherence among female adolescents when feeling sick. Future studies may elucidate how gender and gender responsibilities influence the doctor-patient relationship in this setting and could form the basis for effective gender-sensitive interventions [24].

### Addressing social factors

The proportion (22%) of adolescents working for pay is concerning and may explain the lower school attendance rate in our study population. Education exerts a positive influence on adolescents living with HIV [24] and is a considerable protective factor against poor mental outcomes, risky sexual behavior and substance abuse [40, 41]. Our findings offer additional rationale to improve school retention by confronting discrimination in schools and normalizing HIV. HIV-related stigma is a primary determinant in adolescent health and is also a decisive motivator in the transition process [42]. In addition, expansion of peer support to the two-thirds of adolescents who did not belong to such groups is needed, as participation in peer support is associated with improved medical adherence [24, 43]. Given that access to available social protection support was suboptimal, our results argue for adolescent-focused interventions at multiple layers of the sociological context [24] to ensure that every needy adolescent has access to available wrap around services to support successful adolescent transition. In addition, future analyses are needed to explore the socioeconomic factors driving these students out of school and into the workforce.

### Addressing health system factors

An inadequate drug supply was common which may affect ART adherence and partially contribute to the high rate of school absences. Improved medication adherence is associated with a daily routine [39], which is reliant upon a consistent supply of medication. Moreover, the transition from pediatric to adult care has the potential for being a significant psychological burden [44] on adolescents living with HIV, which requires adequate supply and adolescent-friendly services, devoid of stigma. In the current context, strengthening supply of ARV will be essential. At the same time, there is a need to strengthen the provision of counselling on transition and enhance adolescent familiarization with adult clinics as part of the transition. Furthermore, despite policies requiring allocation of a transition case manager, only a quarter of adolescents in this study had been allocated one. This will also need to be enhanced in order to support successful transition of adolescents to adult HIV care. Transition managers provide and link adolescents to available social support and protection.

### Limitations

Our study offers a picture of the HIV epidemic among Cambodian adolescents and the sociocultural phenomena they experience while transitioning from pediatric to adult HIV care. However, and despite our sampling methodology, the generalizability of our results to the entire population of adolescents living with HIV in Cambodia may be limited. Potential for bias due to social desirability or recall biases were minimized by validating responses against medical records whenever possible, but may not have been eliminated.

## Conclusions

The considerable advancements made in reducing new infections from mother-to-child transmission indicates that a focused worldwide effort, robust political commitment and leadership at the national level can produce substantial results. The same type of concentrated focus must now be shifted to adolescents. Our study reports that social protection mechanisms are reaching a number of adolescents living with HIV in need, but others remain without support. Multi-sectoral interventions, boosting school attendance, mitigating HIV-related stigma, and expanding coverage of social protection mechanisms are necessary to improving the health and quality of life of all adolescents living with HIV in Cambodia.

## References

[1] Patton GC, Viner RM, le Linh C, Ameratunga S, Fatusi AO, Ferguson BJ (2010). Mapping a global agenda for adolescent health. J Adolesc Health.

[2] Friedman HL (1992). Changing patterns of adolescent sexual behavior: consequences for health and development. J Adolesc Health.

[3] Viner RM, Ozer EM, Denny S, Marmot M, Resnick M, Fatusi A (2012). Adolescence and the social determinants of health. Lancet.

[4] Mburu G, Hodgson I, Teltschik A, Ram M, Haamujompa C, Bajpai D (2013). Rights-based services for adolescents living with HIV: adolescent self-efficacy and implications for health systems in Zambia. Reprod Health Matters..

[5] Idele P, Gillespie A, Porth T, Suzuki C, Mahy M, Kasedde S (2014). Epidemiology of HIV and AIDS among adolescents: current status, inequities, and data gaps. J Acquir Immune Defic Syndr.

[6] Mburu G, Hodgson I, Kalibala S, Haamujompa C, Cataldo F, Lowenthal ED (2014). Adolescent HIV disclosure in Zambia: barriers, facilitators and outcomes. J Int AIDS Soc..

[7] Kim SH, Gerver SM, Fidler S, Ward H (2014). Adherence to antiretroviral therapy in adolescents living with HIV: systematic review and meta-analysis. AIDS..

[8] Nachega JB, Hislop M, Nguyen H, Dowdy DW, Chaisson RE, Regensberg L (2009). Antiretroviral therapy adherence, virologic and immunologic outcomes in adolescents compared with adults in southern Africa. J Acquir Immune Defic Syndr.

[9] Cruz ML, Cardoso CA, Darmont MQ, Souza E, Andrade SD, D’Al Fabbro MM (2014). Viral suppression and adherence among HIV-infected children and adolescents on antiretroviral therapy: results of a multicenter study. J Pediatr.

[10] Zanoni BC, Mayer KH (2014). The adolescent and young adult HIV cascade of care in the United States: exaggerated health disparities. AIDS Patient Care STDS..

[11] Busza J, Strode A, Dauya E, Ferrand RA (2016). Falling through the gaps: how should HIV programmes respond to families that persistently deny treatment to children?. J Int AIDS Soc..

[12] Fernet M, Wong K, Richard ME, Otis J, Levy JJ, Lapointe N (2011). Romantic relationships and sexual activities of the first generation of youth living with HIV since birth. AIDS Care..

[13] Fair C, Albright J (2012). “Don’t tell him you have HIV unless he’s ‘the one’”: romantic relationships among adolescents and young adults with perinatal HIV infection. AIDS Patient Care STDS..

[14] Binagwaho A, Fuller A, Kerry V, Dougherty S, Agbonyitor M, Wagner C (2012). Adolescents and the right to health: eliminating age-related barriers to HIV/AIDS services in Rwanda. AIDS Care..

[15] Judd A, Sohn AH, Collins IJ (2016). Interventions to improve treatment, retention and survival outcomes for adolescents with perinatal HIV-1 transitioning to adult care: moving on up. Curr Opin HIV AIDS..

[16] Ben-Farhat J, Schramm B, Nicolay N, Wanjala S, Szumilin E, Balkan S (2017). Mortality and clinical outcomes in children treated with antiretroviral therapy in four African vertical programs during the first decade of paediatric HIV care, 2001–2010. Trop Med Int Health..

[17] Bryant M, Beard J (2017). Orphans and vulnerable children affected by human immunodeficiency virus in sub-Saharan Africa. Trop Med Int Health..

[18] Andrews G, Skinner D, Zuma K (2006). Epidemiology of health and vulnerability among children orphaned and made vulnerable by HIV/AIDS in sub-Saharan Africa. AIDS Care..

[19] Brown LK, Lourie KJ, Pao M (2000). Children and adolescents living with HIV and AIDS: a review. J Child Psychol Psychiatry.

[20] Schenk KD, Michaelis A, Sapiano TN, Brown L, Weiss E (2010). Improving the lives of vulnerable children: implications of Horizons research among orphans and other children affected by AIDS. Public Health Rep.

[21] Midtbo V, Shirima V, Skovdal M, Daniel M (2012). How disclosure and antiretroviral therapy help HIV-infected adolescents in sub-Saharan Africa cope with stigma. Afr J AIDS Res..

[22] Hodgson I, Ross J, Haamujompa C, Gitau-Mburu D (2012). Living as an adolescent with HIV in Zambia—lived experiences, sexual health and reproductive needs. AIDS Care..

[23] Menon A, Glazebrook C, Campain N, Ngoma M (2007). Mental health and disclosure of HIV status in Zambian adolescents with HIV infection: implications for peer-support programs. J Acquir Immune Defic Syndr.

[24] Mburu G, Ram M, Oxenham D, Haamujompa C, Iorpenda K, Ferguson L (2014). Responding to adolescents living with HIV in Zambia: a social-ecological approach. Child Youth Serv Rev..

[25] Cluver LD, Toska E, Orkin FM, Meinck F, Hodes R, Yakubovich AR (2016). Achieving equity in HIV-treatment outcomes: can social protection improve adolescent ART-adherence in South Africa?. AIDS Care..

[26] Lowenthal ED, Bakeera-Kitaka S, Marukutira T, Chapman J, Goldrath K, Ferrand RA (2014). Perinatally acquired HIV infection in adolescents from sub-Saharan Africa: a review of emerging challenges. Lancet Infect Dis..

[27] Lee S, Hazra R (2015). Achieving 90-90-90 in paediatric HIV: adolescence as the touchstone for transition success. J Int AIDS Soc..

[28] Kakkar F, Van der Linden D, Valois S, Maurice F, Onnorouille M, Lapointe N (2016). Health outcomes and the transition experience of HIV-infected adolescents after transfer to adult care in Quebec, Canada. BMC Pediatr..

[29] Ryscavage P, Macharia T, Patel D, Palmeiro R, Tepper V (2016). Linkage to and retention in care following healthcare transition from pediatric to adult HIV care. AIDS Care..

[30] Wiener LS, Kohrt BA, Battles HB, Pao M (2011). The HIV experience: youth identified barriers for transitioning from pediatric to adult care. J Pediatr Psychol.

[31] Bygrave H, Mtangirwa J, Ncube K, Ford N, Kranzer K, Munyaradzi D (2012). Antiretroviral therapy outcomes among adolescents and youth in rural Zimbabwe. PLoS ONE.

[32] Westling K, Naver L, Vesterbacka J, Belfrage E (2016). Transition of HIV-infected youths from paediatric to adult care, a Swedish single-centre experience. Infect Dis..

[33] Righetti A, Prinapori R, Nulvesu L, Fornoni L, Viscoli C, Di Biagio A (2015). Transitioning HIV-infected children and adolescents into adult care: an Italian real-life experience. J Assoc Nurses AIDS Care.

[34] Yi S, Ngin C, Pal K, Khol V, Tuot S, Sau S (2017). Transition into adult care: factors associated with level of preparedness among adolescents living with HIV in Cambodia. AIDS Res Ther..

[35] Skinner J (2006). Probability proportional to size (PPS) sampling.

[36] Sharma N, Willen E, Garcia A, Sharma TS (2014). Attitudes toward transitioning in youth with perinatally acquired HIV and their family caregivers. J Assoc Nurses AIDS Care.

[37] Bakeera-Kitaka S, Nabukeera-Barungi N, Nostlinger C, Addy K, Colebunders R (2008). Sexual risk reduction needs of adolescents living with HIV in a clinical care setting. AIDS Care..

[38] World Medical Association (WMA) (2008). Declaration of Helsinki: ethical principles for medical research involving human subjects (2008 Revision).

[39] Dowshen N, D’Angelo L (2011). Health care transition for youth living with HIV/AIDS. Pediatrics.

[40] Magnani RJ, Karim AM, Weiss LA, Bond KC, Lemba M, Morgan GT (2002). Reproductive health risk and protective factors among youth in Lusaka, Zambia. J Adolesc Health..

[41] Shackleton N, Jamal F, Viner RM, Dickson K, Patton G, Bonell C (2016). School-based interventions going beyond health education to promote adolescent health: systematic review of reviews. J Adolesc Health.

[42] Seng SDT, Welle E, Mok S, Soch K, Tep S, et al. Stigma and discrimination as factors affecting the transition from paediatric to adult HIV care services by children living with HIV aged 12–17 in Cambodia: a qualitative exploratory study. In: International AIDS conference Abstract TUPE-286. Melbourne, Australia; 2014.

[43] Mupambireyi Z, Bernays S, Bwakura-Dangarembizi M, Cowan FM (2014). “I don’t feel shy because I will be among others who are just like me…”: the role of support groups for children perinatally infected with HIV in Zimbabwe. Child Youth Serv Rev..

[44] Miles K, Edwards S, Clapson M (2004). Transition from paediatric to adult services: experiences of HIV-positive adolescents. AIDS Care..

